# Theory of the *sp*–*d* coupling of transition metal impurities with free carriers in ZnO

**DOI:** 10.1038/s41598-021-83258-1

**Published:** 2021-02-15

**Authors:** Anna Ciechan, Piotr Bogusławski

**Affiliations:** Institute of Physics, Polish Academy of Sciences, al. Lotników 32/46, 02-668 Warsaw, Poland

**Keywords:** Magnetic properties and materials, Semiconductors, Spintronics, Electronic properties and materials, Magnetic properties and materials, Semiconductors, Electronic structure

## Abstract

The $s,p{-}d$ exchange coupling between the spins of band carriers and of transition metal (TM) dopants ranging from Ti to Cu in ZnO is studied within the density functional theory. The $+U$ corrections are included to reproduce the experimental ZnO band gap and the dopant levels. The *p*–*d* coupling reveals unexpectedly complex features. In particular, (i) the *p*–*d* coupling constants $N_0\beta$ vary about 10 times when going from V to Ni, (ii) not only the value but also the sign of $N_0\beta$ depends on the charge state of the dopant, (iii) the *p*–*d* coupling with the heavy holes and the light holes is not the same; in the case of Fe, Co and Ni, $N_0\beta$s for the two subbands can differ twice, and for Cu the opposite sign of the coupling is found for light and heavy holes. The main features of the *p*–*d* coupling are determined by the *p*–*d* hybridization between the *d*(TM) and *p*(O) orbitals. In contrast, the *s*–*d* coupling constant $N_0\alpha$ is almost the same for all TM ions, and does not depend on the charge state of the dopant. The TM-induced spin polarization of the *p*(O) orbitals contributes to the *s*–*d* coupling, enhancing $N_0\alpha$.

## Introduction

The *s*, *p*–*d* coupling between free carriers and the localized *d*- electrons of the TM dopants constitutes the basic feature of diluted magnetic semiconductors (DMSs)^1,2^. The interest in this class of materials sharply raised after it was demonstrated that the *s*, *p*–*d* coupling enables a control of electronic properties by magnetic field, and vice versa, control of magnetic properties by electric field. Those properties of the DMS-based structures were applied to obtain novel spintronic functionalities. Next, free carriers mediate magnetic interactions between the TM ions in DMSs through the *s*, *p*–*d* coupling, and lead to collective magnetism under appropriate conditions. At the basic level, the electron–electron Coulomb coupling includes the spin-dependent exchange channel, which can be represented in the effective Heisenberg form. In DMSs, the dominant “effective” mechanism of coupling with localized spins of magnetic atoms is different for electrons and holes because of the different symmetries of their wave functions.

Theory of coupling between the conduction *s* electrons and the localized 4*f* shell in the rare earth metals was elaborated by Liu^3^, and assumed to be operative also in the case of the $s{-}3d$ coupling in semiconductors. This mechanism is referred to as direct exchange, because it originates in the direct intra-atomic exchange coupling between the overlapping wave functions of *s* and *d* (or *f*) electrons. In turn, the mechanism of the *p*–*d* coupling proposed by Anderson^4^, i.e., the kinetic exchange, relies on the symmetry-allowed hybridization of the *d*(TM) shell with the hole states, or, in the real space picture, with the *p* orbitals of the anion neighbors of the TM dopant. Both the Liu’s and the Anderson’s models are widely used to study and explain magnetic properties of DMSs^5–10^. In parallel, the *s*, *p*–*d* coupling was evaluated with the density functional theory (DFT) calculations for III–V and II–VI semiconductors^11–15^. This approach treats all electrons on the same footing and includes automatically intra- as well as inter-atomic interactions.

In this work, we employ the DFT calculations to study the *s*, *p*–*d* exchange coupling for the TM impurities ranging from Ti to Cu in ZnO. Our study of the entire 3*d* TM series reveals not only the properties of individual dopants, but also trends in the *s*–*d* and the *p*–*d* couplings, assessing their general features. Interpretation of the results based on the analysis of the relevant wave functions reveals the role of the Liu’s and the Anderson’s mechanisms, but first of all it shows the dominant role played by the *p*–*d* hybridization. This latter effect leads to the spin polarization of not only the *p*(O) orbitals of host oxygen ions in the vicinity of the TM ion (leading to the spin splitting of the valence band maximum (VBM) and finite $N_0\beta$s), but also of their *s*(O) orbitals, what provides an additional contribution to the *s*–*d* coupling. In the literature, the *p*–*d* exchange constant $N_0\beta$ is often tacitly assumed to be a constant independent of factors such as the charge state of the dopant. This picture is not compatible with the dominant role of the hybridization, which depends on the inverse energy distance between the *d*(TM)-induced levels and the VBM. Indeed, the pronounced dependence of the TM level energies on its charge state can be reflected not only in the magnitude, but also in the sign of $N_0\beta$. Moreover, $N_0\beta$ can be different for the light and heavy hole subbands depending on the detailed electronic structure of a TM ion.

## Results

### TM impurity levels in ZnO

**Figure 1:**
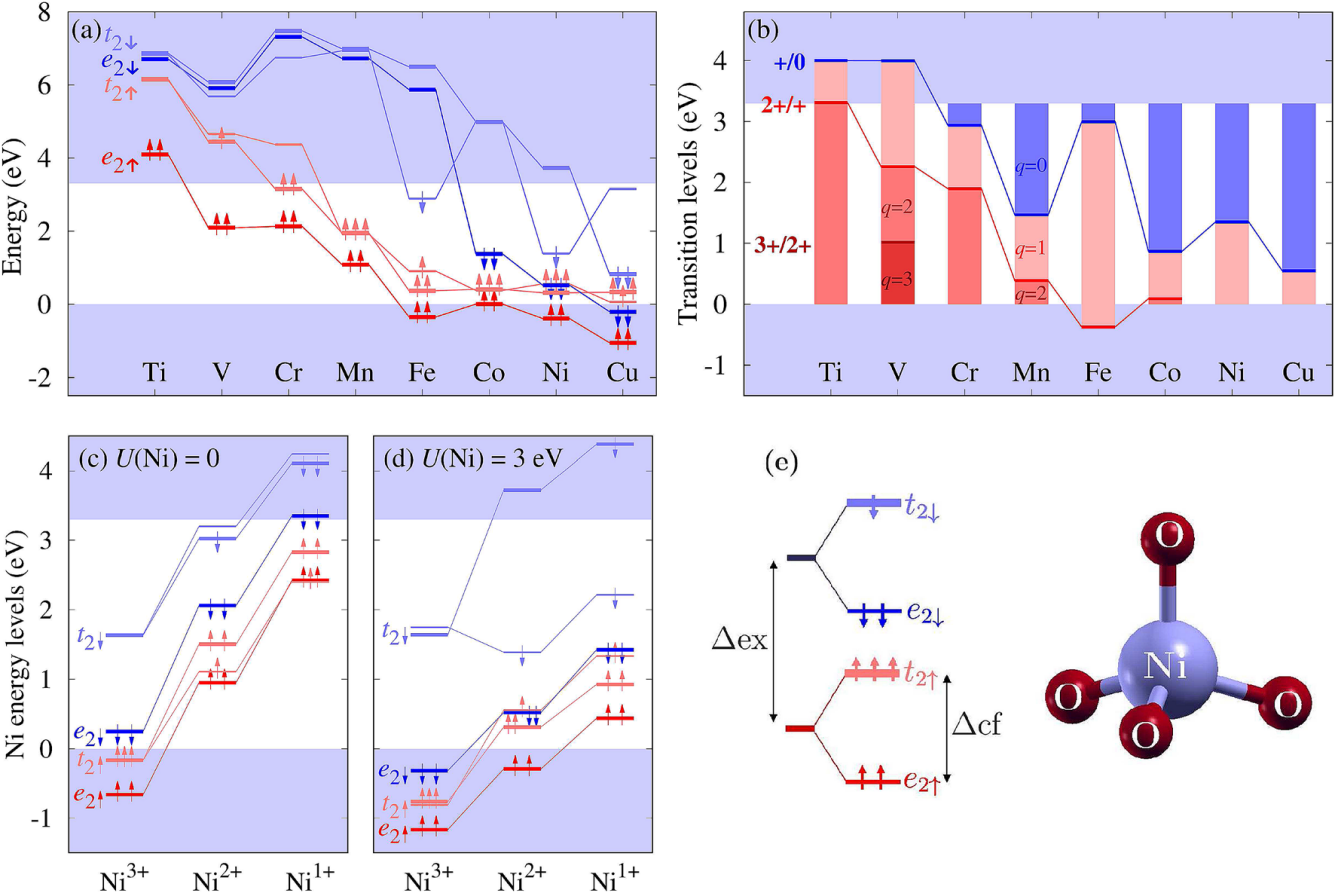
(**a**) Single particle levels of charge neutral TM ($q=0$) and (**b**) transition levels $\varepsilon (q/q')$ of TMs in ZnO obtained with the optimized *U*(TM) values (see Method of calculations). As it is schematically indicated, Fe^4+^ is stable in highly *p*-doped samples. Charge state dependence of the Ni levels for (**c**) *U*(Ni) = 0 and (**d**) *U*(Ni) = 3 eV. In each panel, the levels of TM below the VBM are shown only schematically. (**e**) Schematics of exchange ($\Delta {\mathrm {ex}}$) and tetrahedral crystal field ($\Delta {\mathrm {cf}}$) splittings of Ni^2+^. In the wurtzite structure the three planar bonds are not equivalent with the vertical one, which causes further splitting of triplet levels. Arrows denote spins.

Several magnetic properties of a TM dopant are determined by its energy levels relative to the VBM and the conduction band minimum (CBM). An exemplary band structure of TM-doped ZnO is discussed in Supplementary Information, see Figs. S2 and S3, while the relevant results, necessary to understand the mechanism of the *s*, *p*–*d* coupling, are given in Fig. 1. We first recall that the *d*(TM) shell of a substitutional TM ion in a zinc blende crystal is split into a $e_2$ doublet and a $t_2$ triplet higher in energy. Both states are spin split by the exchange coupling, stronger than the crystal field, and all TM ions in ZnO are in the high spin state, see Fig. 1e. Next, in ZnO the triplets are further split by the uniaxial wurtzite crystal field into singlets and doublets. This holds also for the *p*(O)-derived VBM, which is split by 64 meV into a light hole singlet and a heavy hole doublet, denoted by $A_1$ and $E_2$ in the following.

When the *U*(TM) corrections are employed, the splitting of a $t_2$ state depends on its occupation. The *U*-induced contribution $V_U$ to the Kohn–Sham potential is^16^

1 $$\begin{aligned} V_U|\psi _{k\nu }^\sigma \rangle = U \sum _{m,\sigma } (1/2 - \lambda _m^\sigma )|\phi _m\rangle \langle \phi _m|\psi _{k\nu }^\sigma \rangle , \end{aligned}$$

where $\phi _m$ are the localized *d* orbitals occupied by $\lambda _m^\sigma$ electrons, and $\psi _{k\nu }^\sigma$ are the Kohn–Sham states for the wave vector *k*, band $\nu$, and spin $\sigma$. The $V_U$ potential only acts on the contribution of the *m*th *d*(TM) orbital to the given $(\nu ,k,\sigma )$ state. As a result of the *U*(TM) correction, which favours fully occupied or completely empty orbitals over the partially occupied ones^16^, the splitting of the fully occupied $t_2$ is small, about 0.1 eV, which is close to the crystal field splitting of the VBM. Otherwise, the splitting is more pronounced, and can exceed the crystal field splitting. The calculated energy levels of neutral TM^2+^ ions are shown in Fig. 1a. Considering the series Fe–Cu we see that $t_{2\uparrow }$ is very close to the VBM. Interestingly, this trend reflects the *d*-shell energies of isolated TM atoms, see Supplementary Information, Fig. S1.

Figure 1c,d show the charge state dependence of the energies of the Ni levels as an example. With the increasing occupation of the *d* shell the levels shift to higher energies, which is caused by the increasing intrashell Coulomb repulsion between the *d*(TM) electrons^17–19^. A comparison of Fig. 1c,d obtained with *U*(Ni) = 0 and 3 eV, respectively, visualizes the changes induced by the *U*(TM) term.

The possible stable charge states of TM ions are given by transition levels $\varepsilon$ presented in Fig. 1b. In the absence of additional dopants, a TM ion occurs in the neutral $q=0$ charge state, denoted as TM^2+^, as long as its occupied *d* levels are in the gap. In the presence of donors (acceptors), the charge state can change to $q=-1$ ($q=+1$), *i*.*e*., to TM$^{1+}$ (TM^3+^), or even higher ionized states. Pronounced differences in the consecutive $\varepsilon (q/q')$ energies follow from the strong charge state dependencies shown in Fig. 1c,d. None of defects can act as an acceptor since their $\varepsilon (0/-)$ levels lie above the CBM. In all cases except Ti, two or more charge states can be assumed. The *d*(Ti) levels are above the CBM not only for $q=0$, but also for the +1 and +2 charge state, as reflected by the transition level $\varepsilon (2+/+)$ being above the CBM. Therefore, a spontaneous autoionization of two electrons to the CBM takes place, and Ti occurs only in $q=+2$ charge state, in agreement with experiment^20–22^. Consequently, Ti^4+^ has no *d* electrons, its spin vanishes and so does the *s*, *p*–*d* coupling, and we omit Ti in the following. Also the V impurity has a $\varepsilon (+/0)$ level above the CBM and does not assume the $q=0$ charge state. Electron paramagnetic resonance studies of ZnO:V indicate that the stable charge state of V is $q=+1$^23,24^, in agreement with Fig. 1b. Other defects can occur in $q=0$, +1 and even +2 charge state depending on the Fermi level. Our results are similar to those previously reported^25,26^.

**Figure 2:**
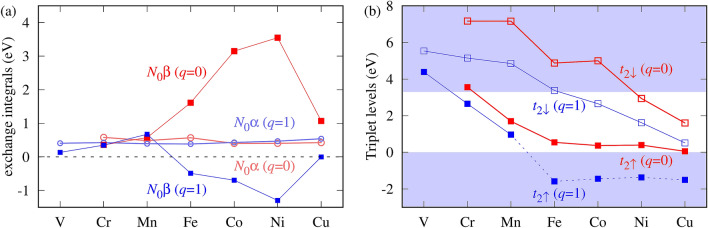
(**a**) The exchange constants $N_0\alpha$ and $N_0\beta$, (**b**) the triplet levels of TM ions in ZnO for both $q=0$ and $q=+1$ charge states. The weighted average values of $N_0\beta$ and $t_{2\sigma }$ are shown. The resonances in the valence band are shown only schematically.

### *s*, *p*–*d* coupling

The coupling of TM ions with the band carriers induces spin splitting of both the CBM, $\Delta \varepsilon _c = \varepsilon _{c\downarrow } - \varepsilon _{c\uparrow }$, and the VBM, $\Delta \varepsilon _{v\gamma } = \varepsilon _{v\gamma \downarrow } - \varepsilon _{v\gamma \uparrow }$. Here, $\gamma$ denotes the $A_1$ or the $E_2$ partner of the VBM. The exchange constants, $N_0\alpha$ for the *s*–*d* coupling and $N_0\beta _{\gamma }$ for the *p*–*d* coupling, are obtained directly from those splittings for supercells containing a single TM ion^12^. Since the splittings of the light hole $A_1$ and the heavy hole $E_2$ bands are different, we have

2 $$\begin{aligned}&N_0\alpha =\Delta \varepsilon _c/(x \langle S \rangle ), \end{aligned}$$

3 $$\begin{aligned}&N_0\beta _A=\Delta \varepsilon _{vA}/(x \langle S \rangle ),\quad N_0\beta _E=\Delta \varepsilon _{vE}/(x \langle S \rangle ), \end{aligned}$$

where $\langle S \rangle = (N_{\uparrow } - N_{\downarrow })/2$, the total spin of the supercell, is the difference in the number of spin-up and spin-down electrons, $N_0$ is the density of the cation sites in ZnO, and *x* is the composition of $\hbox {Zn}_{1-x}\hbox {TM}_x\hbox {O}$ ($x=0.028$ for our supercells). Our definitions imply that both $N_0\alpha$ and $N_0\beta$ are positive for the ferromagnetic (FM) and negative for the antiferromagnetic (AFM) coupling of conduction or valence *electrons* with the TM ion. Note that the FM (AFM) coupling for valence electrons implies the FM (AFM) coupling for holes as well.

Figure 2a and Table 1 shows the central result of this paper, namely the calculated exchange constants $N_0\alpha$, $N_0\beta _A$, $N_0\beta _E$ and their average $N_0\beta = (1/3) (N_0\beta _A + 2 N_0\beta _E )$ for the 3*d* TM in ZnO in both $q=0$ and $q=+1$ charge states. One can observe the following features that characterize the results:

(i) The constant $N_0\alpha$ is about 0.5 eV for all TM dopants, and practically does not depend on their charge state. The constant is positive, so the conduction electrons are ferromagnetically coupled with the TM impurities^3^.
(ii) In contrast to $N_0\alpha$, the constant $N_0\beta$ is strongly dependent on the chemical identity of the dopant, as it increases by an order of magnitude from 0.36 eV for Cr^2+^ to 3.55 eV for Ni^2+^.
(iii) $N_0\beta$ can drastically depend on the impurity charge state: while the coupling is FM for neutral centers, it changes the sign to AFM for positively charged Fe, Co, and Ni ions.
(iv) In the case of Cu, the *p*–*d* coupling with the $A_1$ and $E_2$ subbands is of the opposite character, resulting in opposite sign of the corresponding $N_0\beta _\gamma$, see Table 1.

**Table 1:** Calculated exchange constants $N_0\alpha$, $N_0\beta _A$ and $N_0\beta _E$ and their average $N_0\beta$ (in eV) of TM^2+^ and TM^3+^ in ZnO.

TM	$q=0$						$q=1$						
	Cr	Mn	Fe	Co	Ni	Cu	V	Cr	Mn	Fe	Co	Ni	Cu
$N_0\alpha$	0.58	0.48	0.56	0.40	0.41	0.43	0.41	0.42	0.39	0.38	0.43	0.47	0.54
$N_0\beta _A$	0.14	0.60	0.80	4.16	2.32	− 5.38	0.18	0.76	0.48	− 0.62	− 0.91	− 1.73	2.67
$N_0\beta _E$	0.47	0.56	2.02	2.64	4.16	4.29	0.11	0.14	0.77	− 0.42	− 0.59	− 1.08	− 1.34
$N_0\beta$	0.36	0.58	1.61	3.15	3.55	1.07	0.13	0.35	0.67	− 0.49	− 0.69	− 1.30	0.00

## Discussion

### Spin splittings and the exchange-correlation potential in DFT

Before a detailed discussion we present a few general remarks on the *s*, *p*–*d* coupling. When the electron gas is spin polarized, the exchange- correlation potential $V_{xc\sigma }$ depends on the direction of the electron spin $\sigma$. The spin splitting of the CBM is given by

4 $$\begin{aligned} \Delta \varepsilon _c = \varepsilon _{c\downarrow } - \varepsilon _{c\uparrow } =< CBM \downarrow | H^{KS}_\downarrow | CBM \downarrow> - < CBM\uparrow | H^{KS}_\uparrow | CBM \uparrow >, \end{aligned}$$

where $H^{KS}_{\sigma }$ is the Kohn–Sham hamiltonian with the appropriate $V_{xc\sigma }$. An analogous expression holds for $\Delta \varepsilon _v$. To simplify the discussion, we use two approximations. First, we assume that the orbital parts of the CBM wave functions for both spin directions are equal. (Validity of this approximation is discussed below.) Second, for illustrative purposes, we refer to the simple $X\alpha$ approximation, according to which the spin-dependent exchange potential $V_{xc\sigma }$ is given by $V_{xc\sigma }(r) = A [n_{\sigma }(r)]^{1/3}$, where $n_{\sigma }(r)$ is the density of electrons with the spin $\sigma$ and $A=-2 e^2(3/4\pi )^{1/3}$^27^. With those assumptions, Eq. (4) simplifies to

5 $$\begin{aligned} \Delta \varepsilon _c = < CBM | \Delta V_{xc} | CBM >\qquad {\mathrm {with }}\qquad \Delta V_{xc} = V_{xc\downarrow } - V_{xc\uparrow } = A(n_{\downarrow } ^{1/3} - n_{\uparrow } ^{1/3}) \end{aligned}$$

and the potential difference $\Delta V_{xc}$ determines the spin splitting of the CBM.

**Figure 3:**
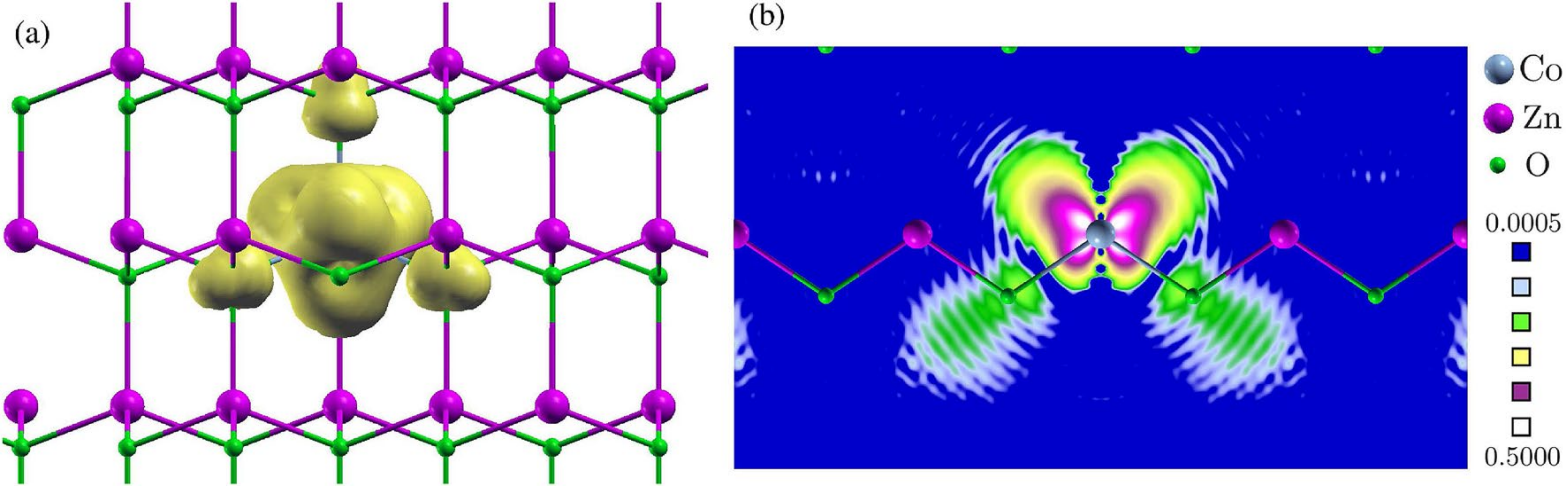
(**a**) Spin density, $n_\uparrow -n_\downarrow$, of ZnO with Co^2+^ (the isosurface value is 0.002 electron/Bohr$^3$) and (**b**) two-dimensional plot of $n^{1/3}_\uparrow - n^{1/3}_\downarrow$ in the plane containing Co–O bonds.

According to Eq. (5), the spin splitting $\Delta \varepsilon _c$ is given by the product of $\Delta V_{xc}$ and the wave function squared. An insight into the mechanisms of the *s*, *p*–*d* coupling is obtained by inspecting those quantities. The first factor, $\Delta V_{xc}$, is a functional of the densities $n_{\sigma }$, and stems from the non-vanishing spin density $\Delta n= n_{\uparrow } - n_{\downarrow }$. It is mainly localized on the *d*-shell of the TM ion. The final charge and spin densities can be considered as a result achieved in two steps. First, in the absence of coupling between the ZnO host and, e.g., the Mn dopant, the ZnO:Mn system consists in the ZnO host with one vacancy and the Mn atom. The total spin of this system is 5/2. After switching on the coupling, *i*.*e*., the *p*–*d* hybridization, the Mn spin “spills” onto the ZnO host, mainly onto the first O neighbors of Mn. Thus, the Mn spin is somewhat reduced and the O neighbors become spin polarized, but the total spin is conserved and equal 5/2. The final spin polarization acts as a source of an additional attractive potential for the spin-up electrons, which determines the FM character of the coupling and causes the spin splitting of the band states.

$\Delta n$ is shown in Fig. 3a for Co in ZnO. It is dominated by the Co orbitals, and also contains a contribution from the spin polarized O nearest neighbors. Figure 3b shows the difference ($n_{\uparrow } ^{1/3} - n_{\downarrow } ^{1/3}$), to which $\Delta V_{xc}$ is approximately proportional. The shape of the isosurface has a tetrahedral symmetry to a good approximation, and reflects that of $\Delta n$. Co^2+^ has 7 *d*-electrons. From Fig. 1a it follows that the two $e_2$ orbitals are occupied with 4 electrons, two with spin-up and two with spin-down, and their total spin is zero. The spin density shown in Fig. 3 is thus dominated by the three $t_{2\uparrow }$ orbitals. Because $V_{xc\sigma }$ is mainly given by the (1/3) power of $n_{\sigma }$, $\Delta V_{xc}$ is smoother and somewhat more delocalized than the spin density, which enhances the role of the O neighbors.

**Figure 4:**
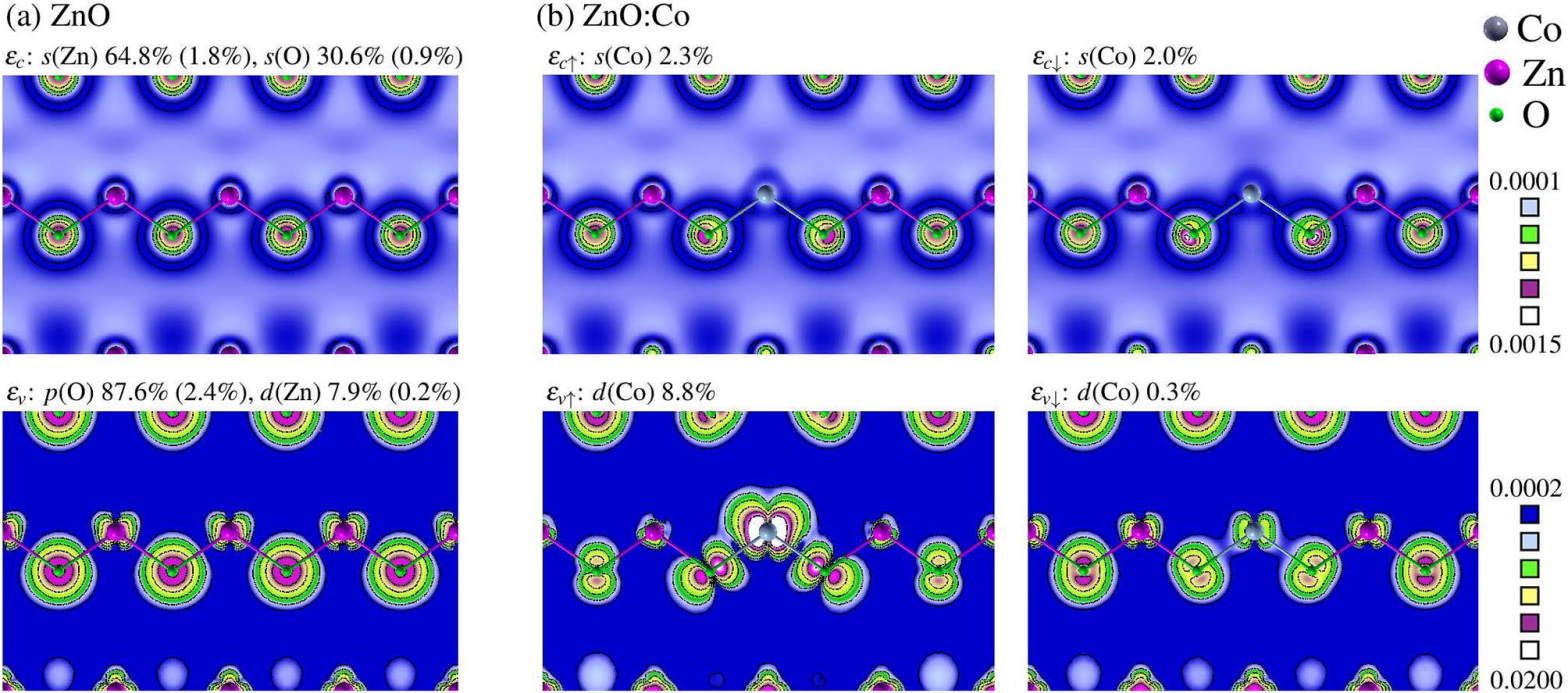
(**a**) The wave functions squared of the CBM and the VBM (sum over *p* states) of ZnO and their orbital compositions. The numbers give the total contribution of orbitals, and in parentheses we give the contribution per one cation or one anion in the 72-atom supercell (e.g., $1.8\% \times 36 = 64.8\%$). (**b**) The wave functions squared of the CBM and the VBM of ZnO:Co^2+^. Left (right) panels show the spin-up (spin-down) states. The contributions of *s*(Co) or *d*(Co) orbitals are shown in each case.

The second factor which determines the spin splitting of a given state is its wave function. In a zinc blende crystal, the CBM of the $\Gamma _1$ symmetry contains the *s* orbitals only, while the $\Gamma _{15}$ VBM states are composed of both *p* and *d* orbitals. In the wurtzite ZnO these selection rules are relaxed by the hexagonal part of the crystal field, but this small effect can be neglected in the discussion. Those features are seen in Fig. 4a, which presents the wave functions of pure ZnO as a reference. In the case of ZnO:Co, Fig. 4b, hybridization between the TM and the host ZnO states results in the contribution from *s*(TM) to the CBM, and from *d*(TM) to the VBM, as it is discussed in detail below. Note that the effect of *p*–*d* hybridization on the $t_{2\sigma }$ and $e_{2\sigma }$ gap states is even more pronounced. It is reflected in their strong delocalization shown in Fig. S5 of Supplementary Information, especially when compared with the compact spin polarization $\Delta n$ shown in Fig. 3.

Our results provide a good illustration of the fact that the Heisenberg form of the coupling, $H_{ex}=-J\ \mathbf{S\cdot s}$, has an “effective” character. Indeed, the Heisenberg hamiltonian suggests that the exchange interaction acts on the spin component of the wave function. Actually, this is not the case, because $V_{xc\sigma }$ is spin-dependent but it acts on the orbital parts of the wave function. It leads to the differences between the spin-up and spin-down partners of CBM and VBM, and the main difference relies in the different contribution of the *d*(TM) to the wave functions shown in Fig. S4 of Supplementary Information.

### *s*–*d* exchange coupling

Theory of the exchange coupling between the *s*-like conduction electrons and the localized *f*-shell in rare earths (REs) was developed by Liu^3^. He considered a restrained set of states, namely the *f*-shell of the RE atom and the CBM, and applied the Hartree–Fock approach to obtain the exchange term. This direct (or potential) exchange stems from the overlap of the corresponding wave functions, and is FM. Because of the strong localization of the *f* orbitals, the overlap integrals extend over the volume of the magnetic atom only, and therefore the *s*–*f* coupling is driven by intra-atomic effects. The Liu’s picture was then applied to the *s*–*d* coupling in DMSs in spite of differences between the two systems, which we discuss in the following.

The tight binding picture allows for an intuitive real space interpretation of the results at the atomic level. The CBM wave functions are represented as sums over the appropriate $s_R$ orbitals of atoms at sites *R* in the supercell:

6 $$\begin{aligned} \psi _{\sigma }(r) = \sum _{R} a_{R\sigma } s_R(r-R), \end{aligned}$$

and are normalized to 1 in the supercell volume. The decomposition coefficients $a_{R\sigma }$ are obtained by projection of the calculated $\psi _{\sigma }$ onto the atomic orbitals. Assuming again that the orbital part of the wave function is the same for both spin directions we have an approximate expression

7 $$\begin{aligned} \Delta \varepsilon _c = a_{R=0}^2<s_{TM} | \Delta V_{xc} | s_{TM}> +\sum _{R=NN} a_{R}^2 < s_O | \Delta V_{xc} | s_O > = \Delta \varepsilon _c(TM) + \Delta \varepsilon _c(O_{NN}), \end{aligned}$$

where the sum is limited to the first oxygen neighbors of the TM ion, in agreement with the strong localization of both $\Delta n$ and $\Delta V_{xc}$, see Fig. 3. In this expression the inter-atomic terms are neglected, which is justified by the smallness of the involved overlap integrals, and the spin splitting of the CBM is the sum of atomic-like contributions. The first one, $\Delta \varepsilon _c(TM)$, corresponds to the Liu picture of the *s*–*d* coupling, while the second one, $\Delta \varepsilon _c(O_{NN})$, originates in the spin polarization of the O ions induced by the *p*–*d* hybridization with *d*(TM).

To estimate the first term of Eq. (7) one needs the splitting energy $\Delta _{spin}^{atom}$ of the $s_{TM}$ orbitals of isolated atoms. Unfortunately, as it is explained in the Supplementary Information, $\Delta _{spin}^{atom}$ can be evaluated only for Mn, because there are fundamental problems with obtaining a correct electronic structure of the remaining TM atoms within the DFT. In the case of Mn, the value of the first term of Eq. (7) is obtained under two assumptions. First, the spin polarization is entirely localized on the TM ion, which corresponds to the Liu’s picture. The calculated $\Delta _{spin}^{atom}$(Mn)$=1.0$ eV. Second, the difference between $a_{TM\uparrow }^2=0.025$ and $a_{TM\downarrow }^2=0.018$ is neglected, and the average value $a_{R=0}^2=<a_{TM\sigma }^2>=0.021$ is used instead. This gives $\Delta \varepsilon _c$(Mn)$= a_{R=0}^2 \Delta _{spin}^{atom} = 0.021$ eV, which is about (2/3) of the actual splitting of 0.034 eV. This estimation is likely to represent the upper limit, since $\Delta V_{xc}$ in ZnO is more delocalized, and thus weaker, than in an isolated Mn atom. Moreover, this shows that the second term of Eq. (7), $\Delta \varepsilon _c(O_{NN})$ contributes about 1/3 to the CBM spin splitting. Thus, the *p*–*d* hybridization plays an indirect but non-negligible role in the *s*–*d* exchange coupling, leading to the spin-polarization not only of *p*(O) but also of *s*(O) electrons and thus enhancing the values of $N_0\alpha$. A comparable situation is expected to take place for other TM ions given the similarity in the CBM wave functions and in spin polarization of the O anions.

As follows from Fig. 2a, $N_0\alpha$ is almost independent of the dopant and its charge state, especially when compared with large changes in $N_0\beta$. Actually, the deviations from the average value of 0.5 eV are about 20 per cent. This result can be related with the intra-atomic character of the *s*–*d* coupling. We first note that the definition of $N_0\alpha$ through $\Delta \varepsilon _c=-N_0\alpha \ \mathbf {S\cdot s}$ implies that $N_0\alpha$ describes the coupling between a free carrier and *one* of the *d*(TM) electrons. This in turn is given (in the Hartree–Fock picture) by the exchange overlap integral between the *s*(TM) and *d*(TM) orbitals of the dopant. Those integrals should be similar within the 3*d* series because of the similarity of the involved *s* and *d* states. Also, both *s* and *d* are not expected to strongly depend on the charge state. Second, the decomposition of the CBM wave functions shows that the contributions from the *s*(TM) orbitals to the CBM are similar for all dopants, about 1.5–2.5% (in the 72-atom supercells), with no clear trend regarding the TM identity or charge state. These two factors combined contribute to the obtained weak dependence of $N_0\alpha$ on the dopant.

**Figure 5:**
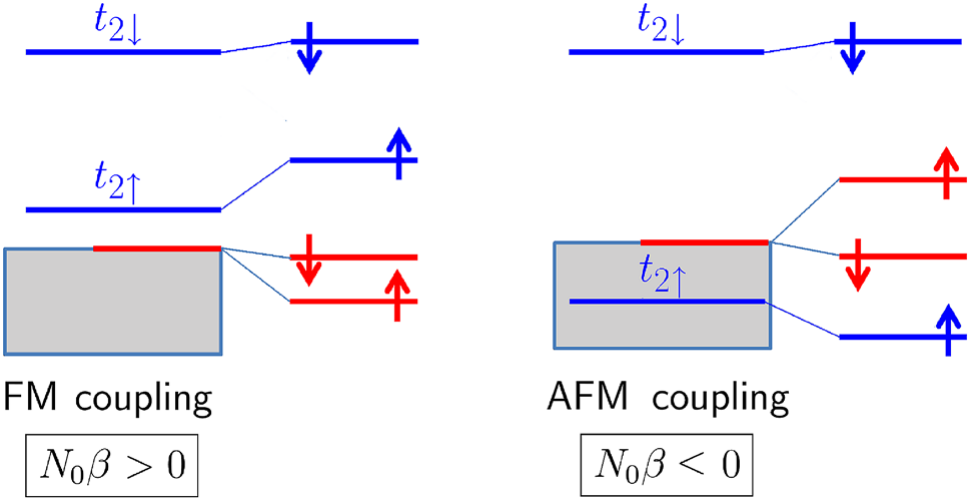
Dependence of the *p*–*d* exchange coupling on the energies of the $t_{2\sigma }$(TM) levels. The hybridization occurs between the states with the same spin, and is spin-dependent. Left panel: both $t_{2\uparrow }$ and $t_{2\downarrow }$ are above the VBM. $t_{2\uparrow }$ is closer to the VBM than $t_{2\downarrow }$, and thus its interaction with the VBM is stronger. The resulting spin splitting corresponds to the FM *p*–*d* exchange coupling. Right panel: $t_{2\uparrow }$ is below the VBM, and the $t_{2\downarrow }$ is above the VBM, which results in the AFM coupling. Gray area is the valence band. Arrows indicate spin direction of levels only, and occupations are not shown. Left panel represents e.g. Mn in ZnO, and the right one Mn in CdTe. Crystal field splittings of the triplets are neglected for simplicity.

### *p*–*d* exchange coupling

Hybridization between the VBM and the *d*(TM) states is essential for the *p*–*d* coupling. It is typically analyzed in the second order of perturbation theory, in which $\Delta \varepsilon _v$ of a cubic semiconductor is determined by the energies of the TM levels $\varepsilon (t_{2\sigma })$ relative to the VBM^15^

8 $$\begin{aligned} \Delta \varepsilon _v = \frac{1}{2}\left( {|V_{hop,\uparrow }|^2\over \varepsilon (t_{2\uparrow }^0) - \varepsilon _v^0}-{|V_{hop,\downarrow }|^2\over \varepsilon (t_{2\downarrow }^0) - \varepsilon _v^0} \right) , \end{aligned}$$

where superscript indexes “0” mean the unperturbed *d* and VBM level energies, and $V_{hop,\sigma }$ is a spin dependent hopping integral between a TM ion and its neighbors. Average energies of the TM triplet levels are shown in Fig. 2b. Although the Figure presents the final self- consistent energies $\varepsilon (t_2)$ rather than $\varepsilon (t_2^0)$, they can serve as a basis for discussion. Equation (8) together with Fig. 2b qualitatively explain the calculated characteristics of $N_0\beta$ in terms of the *d*-shell energy levels relative to the VBM. For the neutral TM dopants, the first term of Eq. (8) gives a positive (*i*.*e*., FM), while the second one gives a negative (AFM) contribution to $N_0\beta$. The first term is dominant, and $N_0\beta$ is positive, since $t_{2\uparrow }$ is closer to the VBM than $t_{2\downarrow }$. With the increasing atomic number, $N_0\beta$ increases due to the decreasing energy denominators, see Fig. 2a. In turn, the $t_{2\uparrow }$ level for positively charged Fe, Co and Ni is *below* the VBM and thus both terms of Eq. (8) lead to AFM exchange coupling. The underlying mechanism is schematically shown in Fig. 5.

**Figure 6:**
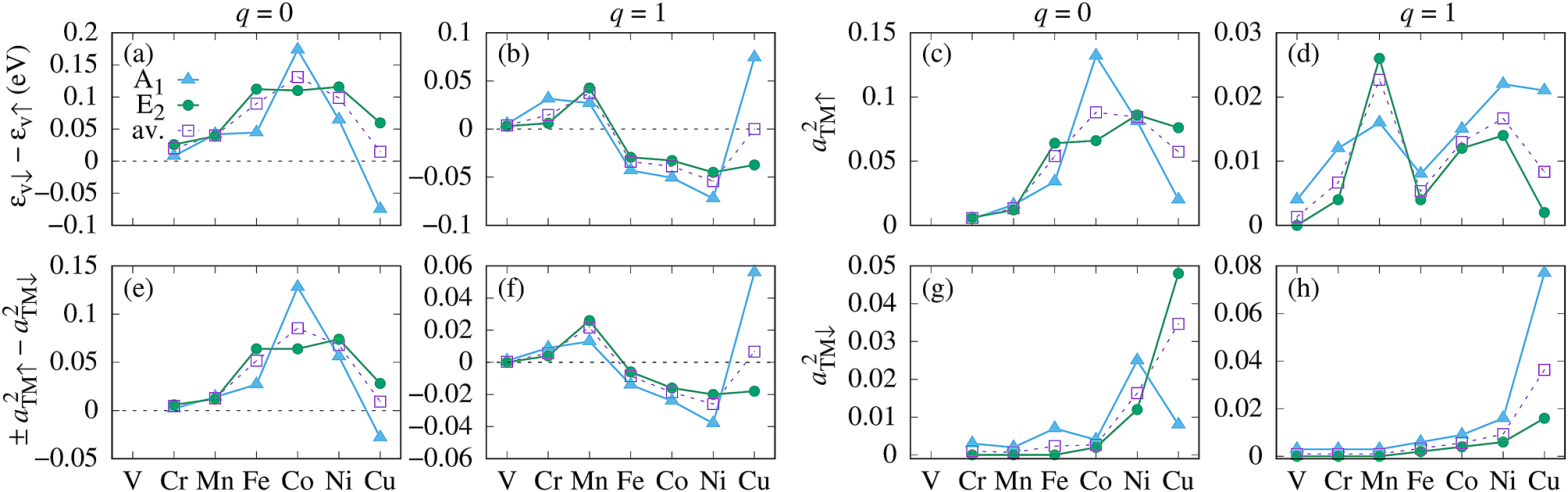
(**a**,**b**) The spin splitting of the singlet $A_1$ and the doublet $E_2$ hole states together with its weighted average, (**c**,**d**) and (**g**,**h**) the appropriate decomposition coefficients $a_{TM\uparrow }^2$ and $a_{TM\downarrow }^2$ of the VBM functions and (**e**,**f**) their combination $\pm a_{TM\uparrow }^2-a_{TM\downarrow }^2$, see details in the text. Charge states *q* are indicated on the top.

In the case of ZnO, the situation is somewhat more complex, since the VBM is a quasi-triplet formed by the $A_1$ and the $E_2$ hole subbands. The corresponding TM-induced spin splitting energies are given in Fig. 6a, b. According to our results, $\Delta \varepsilon _A$ and $\Delta \varepsilon _E$ can substantially differ, and the difference critically depends on both the dopant and its charge state. In particular, the spin density of the Mn^2+^ with the fully occupied spin-up *d*-shell is a fully symmetric $\Gamma _1$ object, it acts on $A_1$ and $E_2$ in a very similar fashion, and consequently $\Delta \varepsilon _A$ and $\Delta \varepsilon _E$ are almost equal. This is not the case of other TM ions. They are characterized by “non-spherical”, *i*.*e*., non-$\Gamma _1$, spin densities, which act differently on the $A_1$ and $E_2$ partners, and induce different $\Delta \varepsilon _A$ and $\Delta \varepsilon _E$. For Cu, even the signs of the splittings are opposite.

The impact of the *p*–*d* hybridization on the *p*–*d* coupling is well illustrated by Fig. 6. The Figure shows the decomposition coefficients $a_{TM\sigma }^2$ of the VBM wave functions in the tight binding picture analogous to Eq. (6). By comparing Fig. 6c,d with g–h one observes that the hybridization is strongly spin-dependent, since the contribution of the spin-up and spin-down TM states to the VBM can differ by as much as one order of magnitude. This stems from the different energies of the TM gap states relative to the VBM, i.e., the different energy denominators in Eq. (8), which also control the mixing of wave functions. In most cases, the spin-down states are more distant from the VBM than the spin-up ones, and the contribution of *d*(TM) to the VBM is appreciably larger for the spin-up than for the spin-down channel. However, since the spin splitting is the energy difference, we have to consider the appropriate combinations of the $a_{TM\uparrow }^2$ and $a_{TM\downarrow }^2$ coefficients rather than their values separately. Depending on the actual level ordering, the combination is $\pm a_{TM\uparrow }^2-a_{TM\downarrow }^2$, consistently with the Eq. (8) , where the $+ (-)$ sign holds when the spin-up TM level is above (below) the VBM. The results are shown in Fig. 6e,f. The very high level of correlation between the splittings $\Delta \varepsilon _A$ and $\Delta \varepsilon _E$ and the contribution of the *d*(TM) orbitals to the VBM is clear. We also note that because of the large differences between $a_{TM\uparrow }^2$ and $a_{TM\downarrow }^2$, the approximate Eq. (7) cannot be applied to the VBM, and the appealing separation into the Liu intra-atomic contribution and the hybridization contribution does not hold. However, the intra-atomic contribution of *d*(TM) to $\Delta \varepsilon _v$ does not vanish. Since the direct exchange leads to the FM coupling, it enhances the hybridization-induced values of the $N_0\beta$s for all TM^2+^ dopants, and reduces the negative values of $N_0\beta$s for Fe^3+^, Co^3+^ and Ni^3+^.

In a complementary approach, one can follow the impact of the *p*–*d* hybridization in the real space picture. The relevant wave functions for ZnO:Co are given in Fig. 4. In agreement with the attractive character of $\Delta V_{xc}$ and with Fig. 6, the spin-up VBM wave function contains a larger contribution of *d*(Co) than its spin-down partner. Indeed, from Fig. 4b it follows that the approximation of Eq. (5) is not justified for the VBM, because the contribution of Co to the spin-up (8.8%) and spin-down states (0.3%) differ over 20 times. The contributions of the *d*(TM) orbitals to the VBM for other TM dopants is shown in Fig. S4 of Supplementary Information. They depend on the dopant and its charge state.

Analyzing the consecutive TM ions we find that, as it follows from Fig. 6, in the case of the light V the contribution from the *d*(TM) shell to the VBM practically vanishes. Accordingly, the VBM spin splittings and the corresponding $N_0\beta$s are small. Next, the average values of $N_0\beta$ increase to 0.4 – 0.6 eV for Cr and Mn with the decreasing energies of $t_2$. In the sequence Fe – Ni, $t_{2\uparrow }$ is very close the valence band, which strongly enhances both the spin splitting of the VBM and $N_0\beta$. Finally, Cu represents an interesting case, since the splittings $\Delta \varepsilon _A$ and $\Delta \varepsilon _E$ have opposite signs. This effect takes place because some of the energies of the *d*(Cu) states are below the VBM, thus changing the sign of the splitting in agreement with Fig. 5.

In the case of charged dopants with $q=+1$, the TM-induced levels are lower in energy than for $q=0$, see Fig. 2b. This leads to higher values of $N_0\beta$ for Cr and Mn. More importantly, in the case of Fe, Co, and Ni, the $t_{2\uparrow }$ levels are $below$ the VBM, which changes the sign of the first term in Eq. (8), and drives the change of character of the *p*–*d* coupling to AFM, as displayed by the negative $N_0\beta$ in Fig. 2a. In particular, the AFM coupling can be expected for Fe, which typically assumes the Fe^3+^ charge state^18^.

### Comparison with previous calculations and with experiment

The role of the *p*–*d* hybridization in the *p*–*d* exchange coupling was recognized early. Typically, it was taken into account by using the Anderson hamiltonian^4^. The calculations employing the Schreiffer–Wolf^5^ transformation were performed and found a negative $N_0\beta$ for Mn in II–VI semiconductors like CdTe^6,11^. Indeed, it was properly recognized that the *d*(Mn) states in CdTe are placed about 2 eV below the VBM. As it is shown in Fig. 5, in this situation the *p*–*d* exchange coupling of holes with the Mn^2+^ spins must be AFM independent of the details of calculations. This approach was used for other TM ions leading to AFM coupling for Mn^2+^, Fe^2+^, Co^2+^^7,9,28^ and FM coupling for Sc^2+^ and Ti^2+^^9,28^. The configuration interaction and cluster-model calculations were also used to evaluate $N_0\beta$ for several II-VI hosts and TM dopants, and to interpret the experimental data; the obtained $N_0\beta$’s were negative indicating the AFM coupling with holes^29–32^. In the papers above, simple expressions for $N_0\beta$ are given, in which the critical factor is the energy of the majority spin *d*(TM) level relative to the VBM. In the case of ZnO, the TM level energies were assumed to be similar to those in CdTe, which leads to AFM coupling with $N_0\beta$ of about $- 3$ eV for Mn^2+^, Fe^2+^ and Co^2+^^8,10,32^.

However, the assumption that $t_{2\uparrow }$(Mn) is below the VBM in ZnO was invalidated by experiment. The measurements^33^ proved that $t_{2\uparrow }$(Mn) is in the band gap, which was subsequently confirmed by both experiment and theory^17,25,34^. Consequently, in this case the *p*–*d* coupling is FM. This should be the case of Cr as well, since $t_{2\uparrow }$(Cr) is higher than that of Mn. Similarly, the energy of the gap levels of Co^2+^ are well established^19,25^, and $N_0\beta >0$ is expected. This may imply that the interpretation of the X-ray data, leading to the negative $N_0\beta$ for Mn^31,32^, and possibly for Fe, Co, and Ni, was not correct. Finally, as observed in Ref.^33^, “location of the *d* levels below the VBM is an essential assumption behind the proposal of mid-gap Zhang-Rice-like states in ZnO:Mn^35^”. According to the results presented above this assumption is not correct.

The same issue, *i*.*e*., correctness of energies of the $t_2$(TM) levels relative to the CBM and VBM, is present in the case of calculations based on the local density approximation. This approximation results in a severe underestimation of the ZnO band gap, and therefore wrong energies of the TM levels and the non-correct sign of the *p*–*d* coupling (e.g., $N_0\beta =-1.81$ eV for Mn in ZnO^14^). On the other hand, when the correct $E_{gap}$ of ZnO is used^25^, the energies of the TM gap states are close to the present results.

We now turn to the experimental results. In early magneto–optical measurements, a strong exchange interaction between band carriers and localized *d*(TM) electrons was reported for Mn, Fe, Co, Ni and Cu^36–39^. By contrast, the *s*, *p*–*d* coupling was not observed so far for Ti, V and Cr ions in ZnO^37^. This latter result is in a reasonable agreement with our findings. Indeed, the stable charge state of Ti is the nonmagnetic Ti^4+^ ($q=2$) as in experiment^20–22^, and thus both exchange constants, $N_0\alpha$ and $N_0\beta$, vanish by definition. Also, V (stable in the V^3+^ charge state according to both experiment^23,24^ and our calculations), as well as Cr, are characterized by small $N_0\beta$ exchange constants.

Subsequent and more detailed experiments were performed for Mn, Fe, Co and Ni. In the case of Mn^2+^, $N_0|\beta -\alpha |=0.2\pm 0.1$ eV was determined by magneto-optical measurements, which gives $N_0\beta =0.5\pm 0.2$ eV or $N_0\beta =0.1\pm 0.2$ eV with the assumption that $N_0\alpha =0.3\pm 0.2$ eV^40^. Similar values, $N_0|\alpha -\beta |=0.1$ eV^41^ and $N_0|\alpha - \beta |=0.16$  eV^42^, were also determined. These results are in a good agreement with the calculated $N_0(\beta - \alpha )=0.1$ eV.

In the case of Co, magnetooptical measurements showed that $N_0|\beta - \alpha |=0.8$ eV^43^. The sign of $N_0\beta$ could not be determined experimentally due to the ambiguity in the valence bands ordering. By assuming $N_0\alpha =0.25$ eV, it was concluded that the *p*–*d* coupling can be either FM with $N_0\beta =1.0$ eV, or AFM with $N_0\beta =-0.55$ eV. Our calculated $N_0\beta$ depends on the charge state. For the neutral Co^2+^ we find the FM coupling ($N_0\beta _\gamma = 4.2$ and 2.6 eV for the $A_1$ and $E_2$ subbands, respectively), while for the positively charged Co^3+^ the coupling is AFM ($N_0\beta _\gamma = -0.9$ and $- 0.6$ eV for the $A_1$ and $E_2$ subbands, respectively). Thus, we obtain that $N_0|\beta -\alpha |=2.6$ eV for Co^2+^, and about 1.2 eV for Co^3+^. The latter value is reasonably close to magnetooptical data^43^.

$N_0\beta$s determined for Fe and Ni are large and negative, namely − 2.7 eV^32^ and $-4.5\pm 0.6$ eV^44^, respectively. These values are somewhat higher than our results for $q=0$ ($N_0\beta _E=2.0$ and $N_0\beta _A=0.8$  eV for Fe, and $N_0\beta _E=4.2$ and $N_0\beta _A=2.3$  eV for Ni), but importantly they are of opposite sign. However, we predict negative but smaller values for those dopants in the $q=+1$ charge states. Here, it should be mentioned that large and negative $N_0\beta$s were proposed also for Mn (–3.0 eV^32^, –2.7^44^) and for Co (–3.4 eV^32^, –2.3^44^). However, as it is pointed out above, interpretation of these measurements is based on particular assumptions regarding the energies of the TM levels, which were subsequently questioned. We conclude that a reliable comparison with experiment for Fe, Co, and Ni requires the charge state of the TM ion to be established.

## Summary and conclusions

Theoretical analysis of the *s*, *p*–*d* exchange coupling between free carriers and the 3*d* transition metal dopants in ZnO was conducted employing the GGA$+U$ method. The present study reveals both the detailed characteristics for each ion, and general trends. A particular care was devoted to reproduce the correct band gap of ZnO and energies of the gap levels of the dopants. The calculated *s*–*d* coupling constant $N_0\alpha$ is about 0.5 eV for all the TM ions, *i*.*e*., it does not depend on the dopant and its charge state. By contrast, the *p*–*d* exchange coupling reveals unexpectedly complex features. First, $N_0\beta$s strongly depend on the chemical identity of the dopant, increasing about 10 times from V to Cu. Second, $N_0\beta$ is different for the two VBM subbands, the light hole $A_1$ and the heavy hole $E_2$, and the corresponding values can differ by a factor 2, or even have opposite signs. Third, not only the magnitude but also the sign of $N_0\beta$ depends on the charge state of the TM ion. In particular, the coupling between holes and Fe, Co and Ni ions in the $q=0$ neutral charge state is strong and ferromagnetic, while for $q=+1$ the coupling changes the character to antiferromagnetic. Finally, the stable charge state of Ti in ZnO is Ti^4+^, in which its spin vanishes.

Analysis of the wave functions reveals how the hybridization between the TM orbitals and the ZnO band states determines the *s*, *p*–*d* exchange coupling. The magnitude of the *p*–*d* coupling is determined by the energies of the *d*(TM) relative to the VBM. The most striking example is that of Cu, for which the $t_{2\uparrow }$(Cu) and VBM are almost degenerate, and the actual ordering of the *d*(Cu)-induced levels and the VBM explains different signs of $N_0\beta$s of the light and the heavy holes, and their large magnitudes. Thus, the *p*–*d*(TM) hybridization leads to the Anderson-like picture of the *p*–*d* coupling, but its role is more complex. In particular, the *p*–*d* hybridization affects not only $N_0\beta$ but also the $N_0\alpha$ constant. The main mechanism of the *s*–*d* coupling is grasped by the Liu’s model, and it originates in the TM intra-atomic exchange interaction between the *s* and *d* electrons. However, the spin polarization of the oxygen neighbors of the TM ion induced by the *p*–*d* hybridization leads to the spin polarization of the *s*(O) orbitals, which contributes about 1/3 to the $N_0\alpha$ constant.

Comparison with experiment is satisfactory for Ti, V, Cr and Mn. In the case of Fe and Co, the definitive conclusions are not possible, because $N_0\beta$ depends on the dopant charge state, which was not assessed in experiment. An acceptable agreement for Co is obtained assuming the Co^3+^ and not the Co^2+^ charge state.

## Method of calculations

The calculations are performed within the density functional theory^45,46^ in the generalized gradient approximation (GGA) of the exchange-correlation potential $V_{xc}$^47^, supplemented by the $+U$ corrections^16^. We use the pseudopotential method implemented in the quantum espresso code^48^, and employ ultrasoft pseudopotentials, which include nonlinear core correction in the case of Co and Ni. The valence atomic configuration is $3d^{10}4s^2$ for Zn, $2s^2p^4$ for O, and $3s^2p^6 4s^2p^0 3d^n$ or $4s^2p^0 3d^n$ for TM ions with *n* electrons on the *d* shell. For V, Ti, Cr, Mn, Fe and Co, the plane-waves kinetic energy cutoffs of 30 Ry for wave functions and 180 Ry for charge density are employed. Convergence was assessed by test calculations with cutoffs of 40 Ry. Following the recommendation of quantum espresso, for Ni and Cu the cutoff is increased to 45 Ry.

Spin-orbit interaction is neglected. We justify this approximation by the results of experiments regarding TM dopants in ZnO. The interaction manifests itself in optical measurements, where the lines of intracenter $d-d$ transitions reveal rich structures, and are split in particular by the spin-orbit coupling. According to the results for Co^49,50^, the spin-orbit splittings of the initial and final states of the T$^4_2$(F) – A$_2$(F) emission can be estimated as 19 and 6 cm$^{-1}$, respectively, which corresponds to 1-3 meV. Similar values, lower than 10 meV, were reported for other TM dopants^50,51^. In the case of Mn^2+^ and Fe^3+^, due to the absence of orbital momentum in the $d^5$ configuration, the second order spin–orbit interaction leads to splitting energies below 1 meV^51^. Moreover, the spin-orbit splitting of the VBM in ZnO is about 10 meV, *i*.*e*., it is very small compared to the spin splittings of the order of 1 eV characterizing heavier atoms and semiconductors such as InSb, CdTe or PbTe. The smallness of the spin- orbit coupling in ZnO:TM justifies its neglect both in our^17–19^ and in the previous ab initio calculations^25,26^.

The electronic structure of the wurtzite ZnO is examined with an $8\times 8\times 8$ *k*-point grid. Analysis of a single TM impurity in ZnO is performed using $3\times 3\times 2$ supercells with 72 atoms, while *k*-space summations are performed with a $3\times 3\times 3$ *k*-point grid. For too small supercells, the spurious defect-defect coupling can distort final results. Convergence of the results with respect to the supercell size was checked for Mn and Co with $6\times 6\times 4$ supercells with 576 atoms. We obtained that the $N_0\alpha$s are the same for both supercells, while $N_0\beta$s are lower by 10 per cent for Mn and 20 per cent for Co in the case of the larger supercell. Ionic positions are optimized until the forces acting on ions became smaller than 0.02 eV/Å.

The parameters $U(\text {Zn})=12.5$ eV for $3d(\text {Zn})$ and $U(\text {O})=6.25$ eV for $2p(\text {O})$ electrons are fitted to reproduce the experimental ZnO band gap $E_{gap}$ of 3.3 eV^52–54^, the width of the upper valence band of 6 eV and the energy of the *d*(Zn)-derived band^55^. Our *U* values are similar to those reported in other works^56–58^. The lattice parameters $a= 3.23$ (3.25) Å, $c = 5.19$ (5.20) Å and $u = 0.38$ (0.38) are underestimated by less than 1 % in comparison with experimental values^59^ given in parentheses.

The used *U* corrections for $3d(\text {TM})$ electrons are: *U*(Ti) = 2.0 eV, *U*(V) = 2.0 eV, *U*(Cr) = 2.0 eV, *U*(Mn) = 1.5 eV, *U*(Fe) = 4.0 eV, *U*(Co) = 3.0 eV, *U*(Ni) = 3.0 eV, and *U*(Cu) = 2.0 eV. For Mn, Fe, Co and Cu they were optimized by a careful fitting to the experimental energies of both intra-center and ionization optical transitions^17–19,60^. For other TM dopants, the *U* corrections are taken to be 2–3 eV, as suggested in the literature^25,26^. We checked that in most cases a variation of the *U*(TM) value by 1 eV alters the impurity levels by about 0.1 eV, and the $N_0\alpha$ or $N_0\beta$ values by less than 0.05 eV. We also mention that in spite of the energetic proximity and strong hybridization between the VBM and the TM-induced levels, a non-ambiguous identification of hole states was always possible based on the analysis of wave functions. However, since the *p*–*d* coupling depends on the inverse energy distance between the TM-induced levels and the VBM, the results are less accurate for Co, Ni and Cu than for Cr and Mn.

Various charge states of the TM dopants are considered. In general, in the absence of additional dopants, a TM ion occurs in the neutral $q=0$ charge state, denoted as TM^2+^, and other charge states *q* can also be assumed when defects are present. Generally, the stable charge state of a defect in a semiconductor depends on the Fermi level. Transition level $\varepsilon (q/q')$ of a defect is defined as the Fermi energy at which the stable charge state changes from *q* to $q'$, or in other words, as the Fermi energy at which formation energies of *q* and $q'$ are equal:

9 $$\begin{aligned} \varepsilon (q/q')=\frac{E(q')-E(q)}{q-q'}-\varepsilon _v, \end{aligned}$$

where *E*(*q*) is the total energy of the doped supercell and $\varepsilon _v$ is the VBM energy of pure ZnO. The finite size effects are taken into account by including the image charge corrections and potential alignment for charged defects^61,62^. The energies $\varepsilon (q/q')$ in the gap determine the possible charge states of TM ions.

Finally, the spin splitting energies of the VBM and CBM are taken directly from the Kohn–Sham levels. Alternatively, they can be obtained from appropriate excitation energies, as discussed in Supplementary Information.

## Supplementary information

Supplementary Information.

## Data Availability

The datasets generated and/or analysed during the current study are available from the corresponding author on reasonable request.
