# Supplementary material for: Theory of the sp–d coupling of transition metal impurities with free carriers in ZnO
Source: Sci Rep. 2021 Feb 15;11:3848. doi: 10.1038/s41598-021-83258-1 (PMC7884780; doi:10.1038/s41598-021-83258-1)
Supplement: Supplementary file 1 — Supplementary Information. [file 41598_2021_83258_MOESM1_ESM.pdf]

Supplementary Information : Theory of the $sp-d$ coupling of transition metal impurities with free carriers in ZnO

Anna Ciechan[1,*] **and Piotr Bogusławski**[1,**]

[1]Institute of Physics, Polish Academy of Sciences, al. Lotników 32/46, 02-668 Warsaw, Poland
[*]ciechan@ifpan.edu.pl
[**]bogus@ifpan.edu.pl

ABSTRACT

Supplement consists of four parts:
TM atoms section treating about single particle, i.e. Kohn-Sham, levels of free TM atoms within DFT
Band structure section devoted to ZnO doped by Mn^{2+}
Wave functions section showing the wave functions squared of CBM, VBM and dopant levels for selected ZnO:TM
$N_0\alpha$ **and** $N_0\beta$ **from excitation energies** section showing alternative way to obtain the spin splitting of the host states

TM atoms

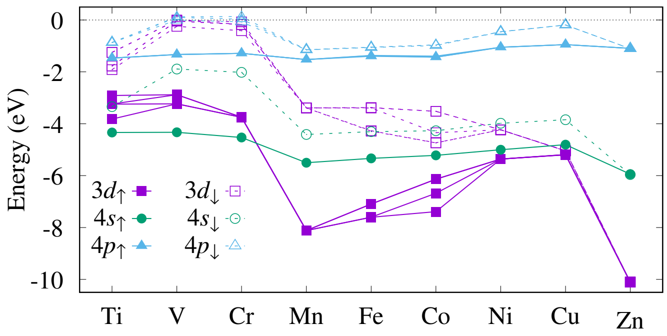

Figure S1. Single particle levels of TM atoms for $q = 0$.

Energies of isolated TM atoms are calculated using cubic supercells with the 20 Å edge, sufficient to decouple adjacent atoms, and the same GGA pseudopotentials and cutoff energies as in the paper.

Calculated properties of the TM dopants in ZnO reflect those of individual TM atoms. Unfortunately, as it was pointed out already by, e.g., Janak[1] the DFT calculations encounter problems when applied to isolated TM atoms, because the self-consistent solutions giving the energy minimum are obtained for fractional occupations of both $3d$ and $4s$ shells. Such configurations are not acceptable based on general arguments, and also they give somewhat distorted $3d$ and $4s$ energies. The issue was discussed for Fe in ZnO.[2] According to our calculations, Mn is the only atom for which the correct integer occupations are obtained, namely d^5s^2 for $q = 0$ and d^5s^1 for $q = +1$ charge state. For the remaining atoms and for both $q = 0$ and $q = +1$, the fractional occupations are found. Our U(TM)= 0 results, shown in Fig. S1, are close to the data obtained by LSDA.[3,4] A characteristic feature is the non-monotonic dependence of the d-shell energies on the atomic number. It explains the non-monotonic dependence of the t_2 and e_2 gap states found for the series Mn–Cu in ZnO. Eigenenergies of the singly ionized $q = +1$ atoms are lower by about 4–5 eV, but this feature persists. Only for $q = +2$ a monotonic dependence takes place.

Band structure

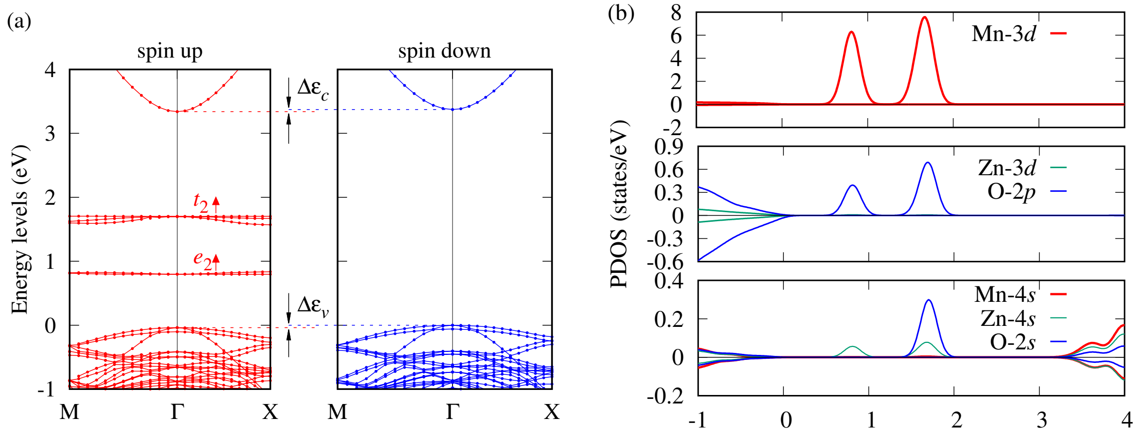

Figure S2. (a) Spin-up and spin-down band structure, and (b) partial density of states of ZnO:Mn^{2+} around the band gap. In (b), densities of one of the oxygen nearest-neighbour of Mn and of one of the next-nearest-neighbor zinc atom of Mn are shown, and thus their contributions to the gap states are relatively large.

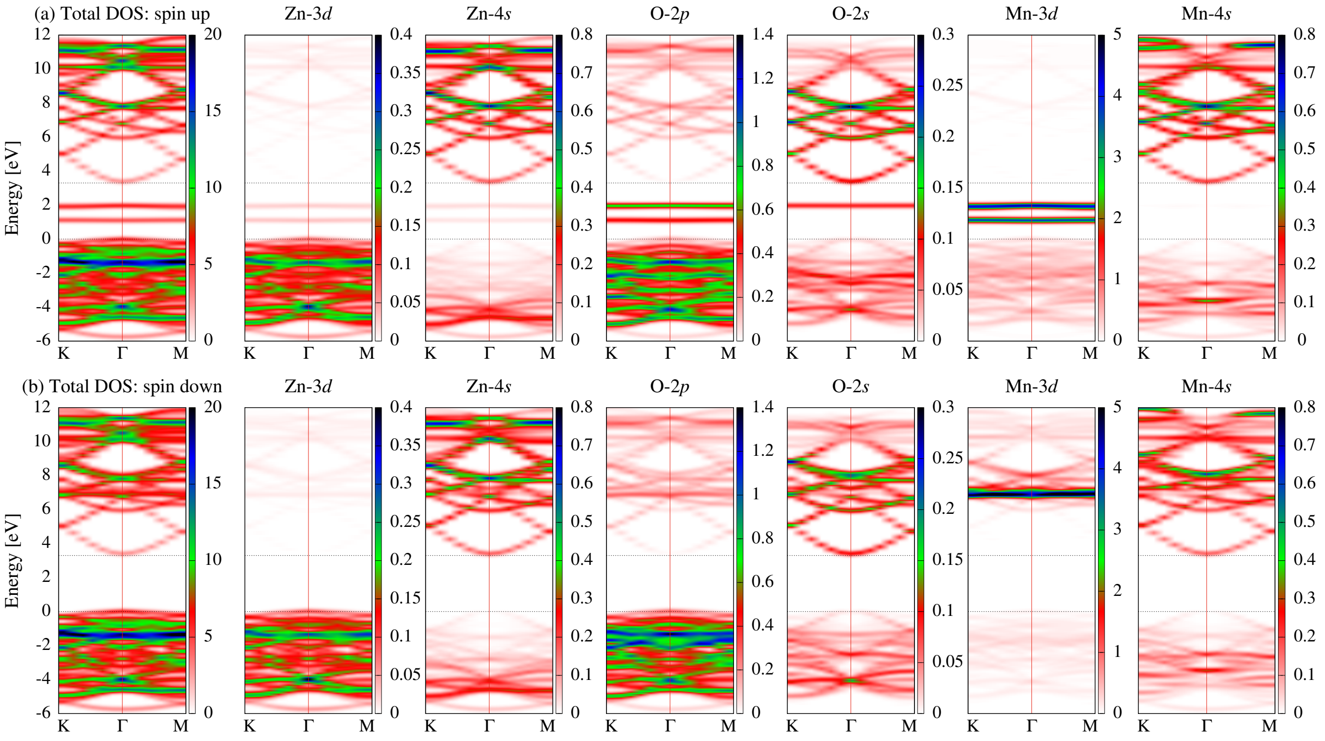

Figure S3. (a) Spin-up and (b) spin-down part of k-resolved partial density of states of ZnO:Mn^{2+}. Note that the color-code scales on the consecutive figures are different. Oxygen and zinc atoms are the same as in Fig. S2.

In Fig. S2 (a), the energy bands of ZnO:Mn^{2+} in the narrow energy window in the vicinity of both the CBM and the VBM are shown, together with their spin splittings. Figure S2 (b) shows the corresponding partial density of states (PDOS) for the spin-up and spin-down channels. Both figures clearly demonstrate that the CBM is composed from s(O) and s(Zn) orbitals with small contribution of s(Mn) (note that PDOSes of single O and Zn atoms are given). In turn, the VBM is composed mainly from p(O) orbitals with an addition of d(Zn). A small contribution of d(Mn) to spin-up valence band is an effect of the $p-d$ hybridization. The hybridization results also in the contribution from O ions to the Mn-induced gap levels (see the wave functions in the next section).

Details of the orbital composition of states are shown in Fig. S3, extending our previous analysis to the whole Brillouin Zone and a large energy window. In particular, the pronounced difference between the DOS of the d(Mn)-up and the d(Mn)-down orbitals shows that $p-d$ hybridization is strongly spin-dependent. The d(Mn)-up contribute mainly to the two spin-up gap states, t_2 and e_2, which form almost dispersionless bands, but this contribution is non-vanishing for all valence bands that extend from 0 to -6 eV, and negligible for the conduction bands. On the other hand, the d(Mn)-down orbitals form a resonance above the CBM. Practically, they contribute only to the conduction bands. The added figures provide a supplementary insight into the $s,p-d$ hybridization and its consequences for $s,p-d$ coupling.

Wave functions

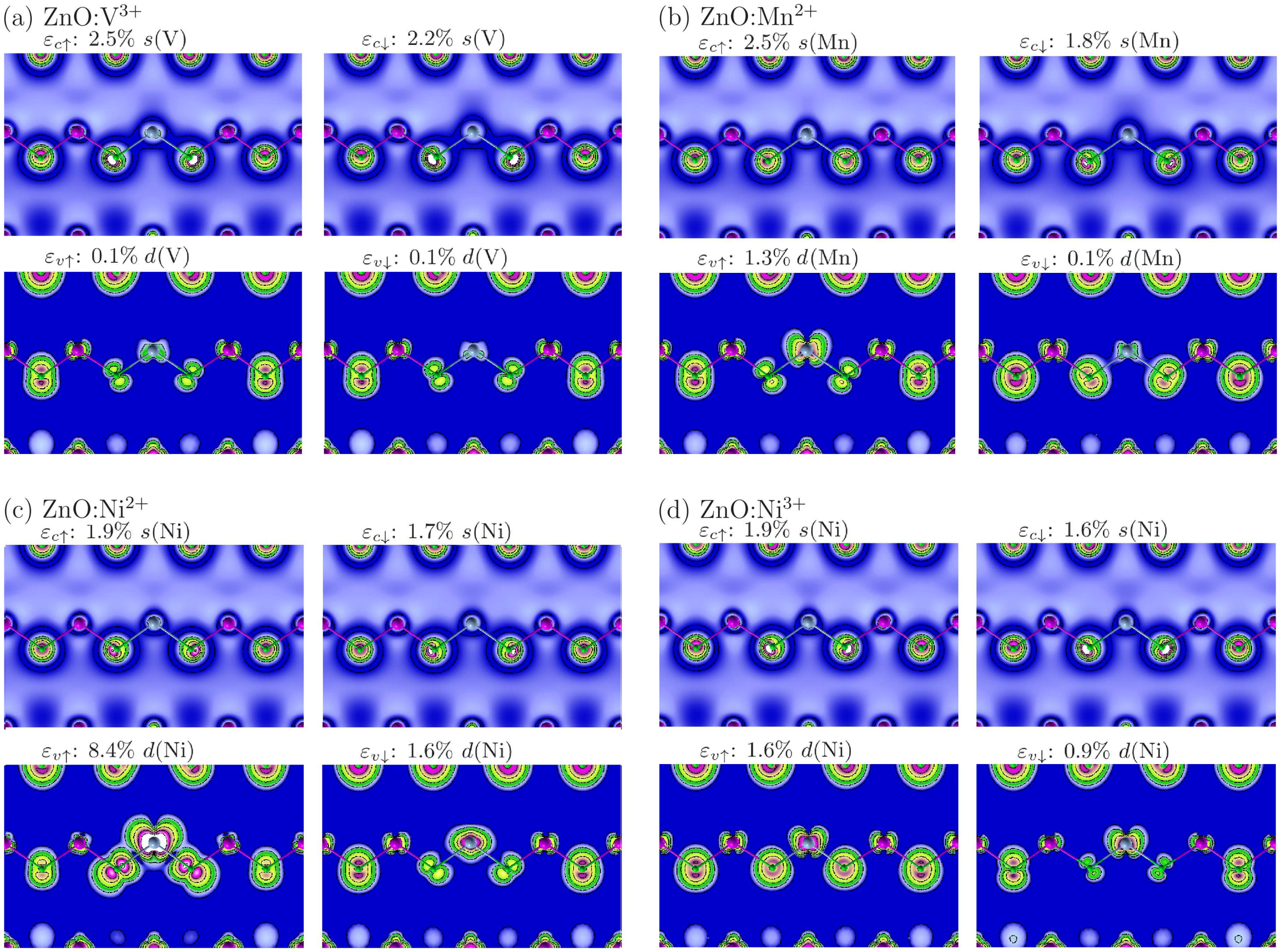

Figure S4. The wave functions squared of the CBM (top panels) and the VBM (bottom panels) for (a) ZnO:V^{3+}, (b) ZnO:Mn^{2+}, (c) ZnO:Ni^{2+} and (d) ZnO:Ni^{3+}. The contribution of TM states is given in each cases.

Figure S4 shows the wave functions of the CBM and VBM for V, Mn and Ni in ZnO. The CBM wave functions are similar for all TM ions, and both spin-up and spin-down partners can be treated as a slightly perturbed CBM of the pure ZnO. In contrast, the VBM states strongly depend on the TM ion and its charge state. The hybridization between the impurity d(TM) and the host p(O) orbitals increases as the energy difference between $t_{2\sigma}$(TM) and the VBM decreases. For V^{3+} with 2 electrons in the d shell, both $t_{2\uparrow}$ and $t_{2\downarrow}$ triplets are empty and lie well above the CBM. Therefore, their contributions to the VBM are comparable and relatively small. The wave functions react locally to an impurity, but the response of the spin-up and -down functions is similar, leading to small $N_0\beta$s. Next, Mn^{2+} (with 5 d electrons) is characterized by the fully occupied $t_{2\uparrow}$ level in the gap and the empty $t_{2\downarrow}$ above the CBM. The $p-d$ hybridization concerns mainly the spin-up channel, which determines the sign and strength of the exchange coupling. Regarding Ni^{2+} with 8 d electrons, the occupied $t_{2\uparrow}$ is very close to the VBM, giving a strong contribution to the $VBM\uparrow$. But the singlet derived from $t_{2\downarrow}$ is also in the gap, and its considerable

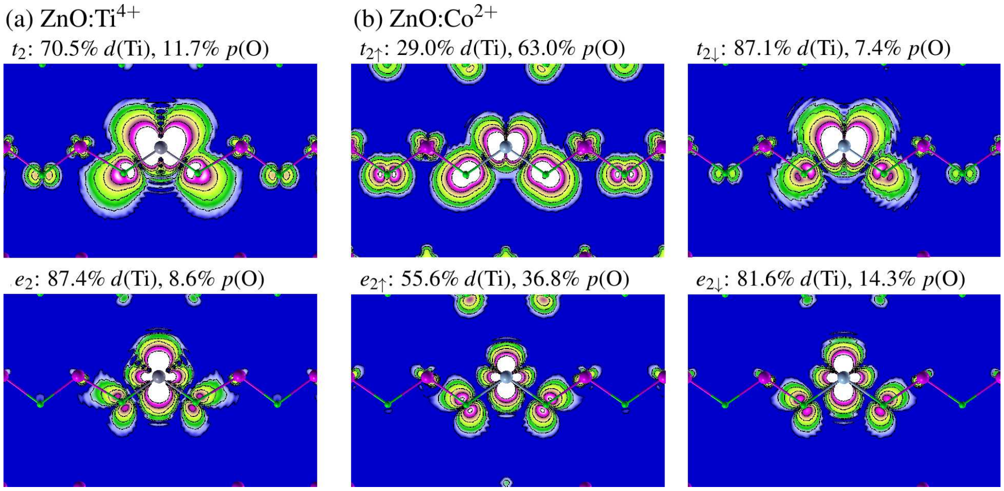

Figure S5. The wave functions squared of the sum of e_2 (top panels) and the sum of t_2 (bottom panels) levels for (a) non-magnetic ZnO:Ti^{4+}, (b) magnetic ZnO:Co^{2+}. The contribution of d(TM) and p(O) states is given in each cases.

hybridization with the VBM effectively reduces $N_0\beta$. A different situation takes place for Ni^{3+} with 7 d electrons. It generates the fully occupied $t_{2\uparrow}$ level below the VBM, and hybridization between those states leads to the negative $N_0\beta$. Moreover, the empty $t_{2\downarrow}$ is in the gap, and its coupling with the $VBM\downarrow$ gives an additional negative contribution to $N_0\beta$.

It is worth to mention that hybridization results in the contribution of p(O) electrons to the TM- induced levels as well. Because of symmetry, the $p-d$ mixing which originates from the VBM states concerns t_2 states, while the contribution of p(O) to e_2 states is an effect of mixing with states below the VBM. As it follows from Fig. S5, the hybridization applies to both magnetic and non-magnetic impurities. However, in the case of the non-magnetic one, like Ti^{4+}, the is no difference between spin-up and spin-down levels. They are characterized by the same energy and the same contribution from p(O) and d(TM) states. Besides, because of Ti levels are much higher in energy than Co levels, the $p-d$ hybridization for Ti is weaker. In turn, for magnetic Co^{2+}, there is a large difference between $p-d$ hybridization for spin-up and spin-down electrons. Finally, one can note a pronounced hybridization-induced delocalization of the TM gap states, especially when compared with the relatively compact spin polarization Δn shown in Fig. 3 of the paper.

$N_0\alpha$ and $N_0\beta$ from excitation energies

The exchange constants of ZnO:TM discussed in the work were calculated directly from the spin splitting of the conduction and the valence bands, see Fig. S2. On the other hand, experimental determination of $N_0\alpha$ and $N_0\beta$ often relies on magnetooptical experiments. The measured energies of excitonic transition can be directly compared with the calculated energies of the excited states of ZnO:TM, which provides an alternative to the approach based on the Kohn-Sham single particle levels used in the paper. To check the consistency of those two approaches, we calculated $\Delta\varepsilon_c$ as a difference in total energy of a supercell with one additional electron at the CBM, either on the spin-up or on the spin-down state. $\Delta\varepsilon_v$ is calculated comparing supercells with one spin-up or spin-down hole at the VBM. The TM charge state is ensured by fixing the occupation numbers of all single particle levels. The comparison was performed for Cr and Mn, for which all occupied dopant levels are well defined in the band gap. Difference in $N_0\alpha$ obtained by both methods is less than 0.01 eV, while that in $N_0\beta$ is less than 0.05 eV, so they can be treated as equivalent.

References

1. Janak, J. F. Proof that $\frac{\partial E}{\partial n_i} = \varepsilon$ in density-functional theory. *Phys. Rev. B* **18**, 7165–7168 (1978).
2. Papierska, J. *et al.* Fe dopant in ZnO: 2+ versus 3+ valency and ion-carrier $s,p-d$ exchange interaction. *Phys. Rev. B* **94**, 224414 (2016).
3. Kraisler, E., Makov, G. & Kelson, I. Ensemble v-representable *ab initio* density-functional calculation of energy and spin in atoms: A test of exchange-correlation approximations. *Phys. Rev. A* **82**, 042516 (2010).
4. Kotochigova, S., Levine, Z. H., Shirley, E. L., Stiles, M. D. & Clark, C. W. Local-density-functional calculations of the energy of atoms. *Phys. Rev. A* **55**, 191–199 (1997).
